# Capnography-guided awake nasal intubation

**DOI:** 10.4103/0019-5049.63639

**Published:** 2010

**Authors:** Gayathri Ramanathan

**Affiliations:** Department of Anaesthesiology, SRM Hospital and Research Centre, Kattangulathur, Chennai, India

Sir,

We read with interest the case report "Capnography guided awake nasal intubation in a 4 month infant with pierre robin syndrome for cleft lip repair: A better technique" by Patra P.[1] It is laudable that Dr. Patra has managed the case efficiently with available resources.

The technique ofcapnography guiding and confirming tracheal intubation has long been taught and practiced. However pediatric fibroscope-assisted intubation is unarguably the technique of choice.

This case report raises a few concerns. The technique of awake blind nasal intubation is challenging even for experienced practitioners. It requires prolonged attempts and results in airway trauma. Neonates and infants sense noxious stimuli such as tracheal intubation. The physiological stress increases the risk of intraventricular brain haemorrhage. Risk factors include hypoxia and hypertension, both of which have been observed in awake intubation.[2] Nasal bleeding can lead to aspiration and endanger life in such infants. Intubation is more difficult in a conscious struggling infant. The author has not specified whether expert help, an airway cart with alternative devices for difficult intubation like Laryngeal mask airway (LMA) and gadgets to performa surgical airway were readily available. This was clearly not an emergency. The assistance of a skilled help, another anaesthetist, is highly valuable and essential in such situations.

Ravishankar *et al*.[3] reported an alternative intubation technique using a rigid nasendoscope and a video camera monitor system in two infants with Pierre-Robin sequence presenting for palatoplasty. In the absence of a flexible paediatric fibrescope, a rigid endoscope (2.7 mm, 70º lateral illumination) was passed orally to provide a view of the glottis on the monitor screen. A tracheal tube, bent into a J-shape using a stylet, was inserted orally and manipulated into the trachea, under video guidance.

We also tried this technique in two cases of difficult intubation in adults. We used a rigid endoscope (4 mm, 45º lateral illumination) passed orally and nasotracheal intubation was performedunder video guidance and spontaneous ventilation. Throughout the procedure a nasopharyngeal airway was passed through the other nostril and O_2_ was administered through a T-piece to prevent hypoxia.

It is a pity that even 150 years after the advent of modern anaesthesia, we are struggling with age-old techniques. Nevertheless, awake intubation is a valuable technique to learn and use when other techniques are inappropriate. Tracheal intubation using a rigid nasendoscope and video camera system proves to be simple technique, permitting a favourable view of the glottis. It should be considered for passing a tracheal tube through the vocal cords in patients who present with a difficult airway.
